# Gender differences in borderline personality disorder: a narrative review

**DOI:** 10.3389/fpsyt.2024.1320546

**Published:** 2024-01-12

**Authors:** Paola Bozzatello, Cecilia Blua, Davide Brandellero, Lorenzo Baldassarri, Claudio Brasso, Paola Rocca, Silvio Bellino

**Affiliations:** Department of Neuroscience, University of Turin, Turin, Italy

**Keywords:** borderline personality disorder, gender differences, treatment, diagnosis, psycopathology, temperament, neuroimaging, symptoms

## Abstract

Borderline personality disorder (BPD) is a severe and complex mental disorder that traditionally has been found to be more frequent in the female gender in clinical samples. More recently, epidemiological studies have provided conflicting data about the prevalence of borderline disorder in the two genders in community samples. In order to explain this heterogeneity, some authors hypothesized the presence of a bias in the diagnostic criteria thresholds (more prevalent in one gender than another), in the population sampling (community versus clinical), in the instruments of evaluation (clinician versus self-report measures), and in the diagnostic construct of BPD. Beyond the question of the different prevalence of the disorder between genders, the debate remains open as to how personality and clinical characteristics, and attitude toward treatments express themselves in the two genders. This narrative review is aimed to provide an updated overview of the differences among genders in BPD in terms of diagnosis, temperamental and clinical characteristics, comorbidities, findings of neuroimaging, and treatment attitudes. Studies that specifically investigated the gender differences in BPD patients are rather limited. Most of the investigations did not consider gender as a variable or were characterized by a significant imbalance between the two genders (more commonly in favor the female gender). The main results indicated that men were more likely to endorse the criteria “intense and inappropriate anger” and “impulsivity,” whereas women endorsed the criteria “chronic feelings of emptiness,” “affective instability,” and “suicidality/self-harm behaviors.” These findings reflect differences in temperament and symptoms of the two genders. Other relevant differences concern pattern of comorbidity, specific neurobiological mechanisms and attitude to treatments. Main limitations were that only one database was searched, time of publications was limited, non-English manuscripts were excluded, and the quality of each paper was not commented.

## 1 Introduction

Borderline personality disorder (BPD) is a complex and heterogeneous mental disease characterized by a pattern of identity diffusion, interpersonal instability, and chronic feeling of emptiness, with episodes of severe affective and impulsive dyscontrol (1).

These psychopathological features are exhibited in both genders, but whether they are more common for men or women is still debated in the literature and remains an open empirical question. Studies that specifically investigated clinical differences among gender in BPD patients are still limited (2). Some of these indicated that certain BPD features are more commonly found in women (3–9) others showed no significant difference across gender (10–13) and the minority of them reported that some clinical manifestations are more common in men (3, 6, 14).

Epidemiological studies have estimated a prevalence of BPD that ranges between 0.7 to 5.8% in the general population (15, 16). In clinical settings this prevalence arises up to 10% of all psychiatric outpatients and up to 15–20% of inpatients (1, 17, 18). Investigations have obtained contradicting results on gender-specific prevalence rates of BPD and the true prevalence of BPD by sex is still unknown. Traditionally, the diagnosis of BPD was considered more common in women, at least in clinical populations (around 75%) (1, 19–22), but recent epidemiological studies reported quite different prevalence rates among countries. In the United States prevalence of BPD did not differ significantly between men and women (5.6% vs. 5.2%) (16).

A Norwegian community sample reported prevalence rates of 0.4% for BPD in men and 0.9% in women (23). By contrast, a study from the United Kingdom found a BPD prevalence rate of 1% in men and 0.4% in women (24).

Some Authors have tried to explain the reasons of this heterogeneity (25–27). Widiger et al. (25) have hypothesized the presence of several biases: in the diagnostic thresholds across disorders more prevalent in one gender or another, in the population sampling (community versus clinical), in the instruments of evaluation (clinician versus self-report measures), and in the diagnostic construct of BPD itself (25). In contrast with the evidence from population-based studies indicating similar rates of BPD among men and women living in the community, BPD is less commonly diagnosed in men in clinical samples (1). Women are known to be more prone to seek help in the health care services for mental health problems compared to men. However, the size of the gender gap could reflect a gender bias in the diagnostic and assessment procedures, leading to under-recognize the disorder in men attending mental health services (27–30). Investigations specifically aimed to evaluate the occurrence of BPD in men are almost totally missing. Some Authors (31) attributed the higher rate of BPD among female gender to the higher risk of sexual abuse among women (early traumatic experiences are known to be considered a relevant risk factor in developing BPD), and to invalidating cultural ideals for women (some unconventional behavior could be considered pathological more often in women than in men). Potential physiopathological explanations of the role of trauma and stress should be considered. In fact, the link between trauma and stress and BPD may be partially mediated by a deregulated hypothalamic–pituitary–adrenal (HPA) axis (32–34) and chronic inflammation (35, 36), affecting brain circules and leading to increase vulnerability to BPD. Given gender differences in trauma/early life stress prevalence these mechanisms are highly relevant.

The majority of the literature on BPD did not consider gender as a separate variable, so there is a scarcity of data concerning potential gender differences in people with BPD that could impact the manifestations, course, and treatment of the disorder in both men and women (7, 13, 37).

The aim of this narrative review is to provide an updated overview of the gender differences in BPD in terms of diagnosis, comorbidity, temperamental and clinical characteristics, neuroimaging and treatment utilization.

**Figure 1:**
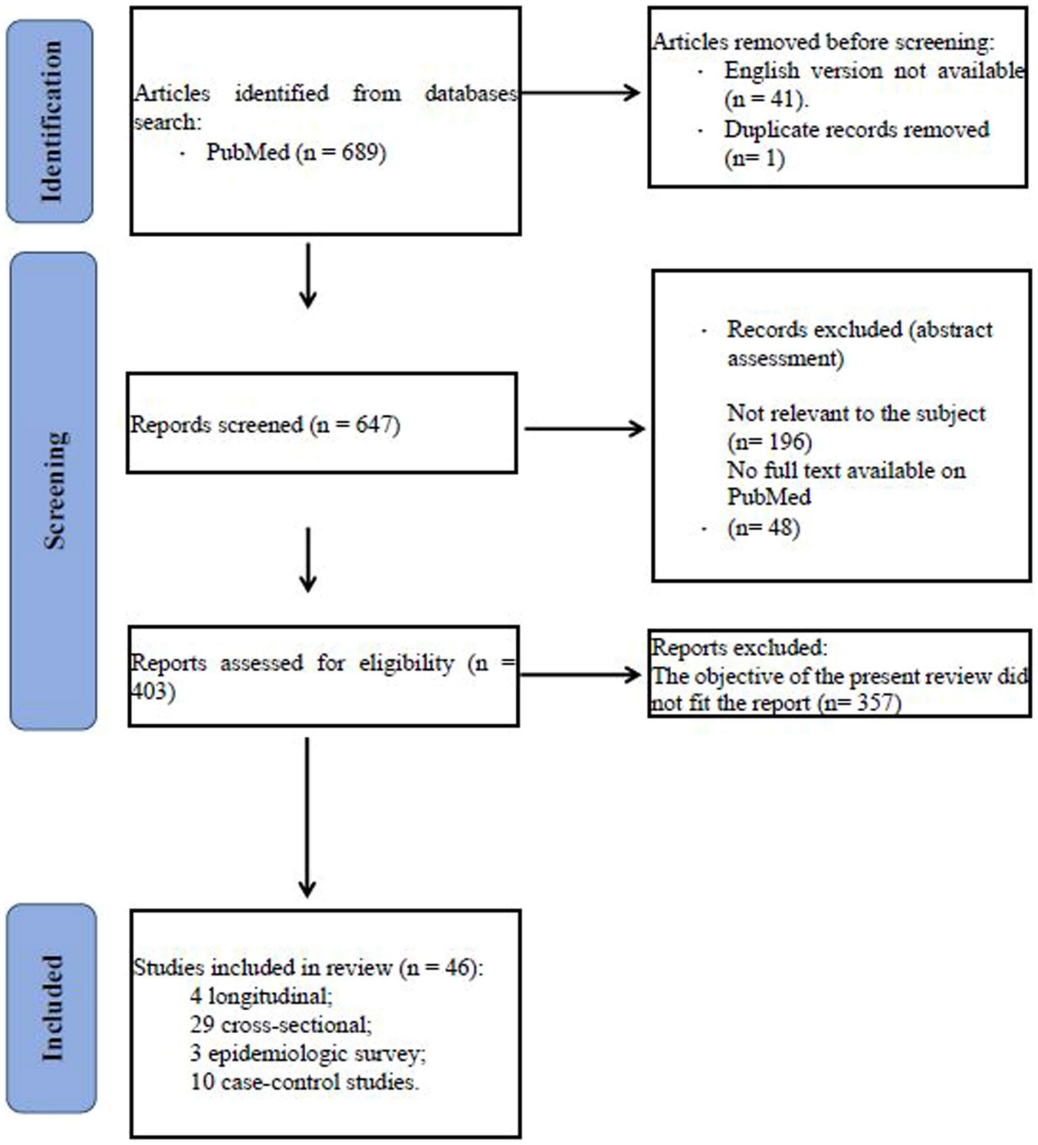
Flowchart.

We conducted a search in Pubmed database of the studies focused on gender differences in borderline personality disorder published between 1983 and 2023 and using the following terms: ((borderline personality disorder) AND ((gender) OR (male gender) OR (female gender))) AND ((temperament) OR (diagnosis) OR (comorbidity) OR (clinical characteristics) OR (neuroimaging) OR (neurobiology) OR (treatment)). Eligibility status for articles was defined by the initial screening of studies on the basis of title and abstract. Papers that passed the initial screening were further selected on the basis of a careful examination of the full manuscript content. The review considered only articles written in English. The literature search is summarized in the flowchart (Figure 1).

We included the following type and number of studies: 4 longitudinal, 29 cross-sectional, 3 epidemiologic survey, 10 case–control studies. Number of studies participants ranged between 13 and 34.653. Age of patients ranged between 11 and 90 years. Most of the participants were Caucasian.

## 2 Gender differences in the diagnosis of BPD

As we have mentioned in the previous paragraph, gender differences in prevalence rates of BPD could be due to the true group differences or might reflect some form of gender bias in diagnostic criteria or in the assessment instruments used to confirm the diagnosis (38).

Kaplan et al. (39) first emphasized the importance of gender bias in DSM psychiatric diagnoses, attributing this bias to the fact that the authors who took part to the DSM-III Task Force were mainly men and had codified certain masculine conceptions of healthy and pathological behaviors (39). Thus women who did not conform to sex-role stereotypes were more likely to receive a psychiatric diagnosis (i.e., BPD) (40).

First studies on this topic investigated whether women were more prone to be diagnosed with BPD than males according to DSM-III criteria (14, 41). The investigation by Henry and Cohen (14) consisted of administering a questionnaire to 277 undergraduate and graduate students without a psychiatric diagnosis. Results showed that healthy men exhibited more characteristics of borderline personality disorder than healthy women. Authors hypothesized that labeling processes may be a contributing factor in the overrepresentation of women among patients who received a diagnosis of BPD. They also stated that there is probably a bias in the diagnostic thresholds: for example “inappropriate and intense anger” was considered more “pathological” in the female sex than in the male, despite being more frequently reported in men. Biases during the diagnostic processes were pointed out in another study (41) aimed to test in 101 clinicians adherence to the diagnostic criteria of personality disorders during the diagnostic process. Findings highlighted a modest concordance between clinical diagnoses and the DSM-Ill criteria for BPD. In particular, authors found that female patients were more likely to be misdiagnosed with BPD when both the physician and the patient were females, suggesting less acceptance of borderline-like traits and behaviors in women by women (41).

The results of another study conducted by the same research group (2002) seem to go in a different direction. Authors enrolled 101 college students who completed a questionnaire (DSM-IV criteria) to describe themselves and to establish for each criterion which would cause difficulty in functioning for females and for males. There were no gender differences in the self-ratings of BPD criteria and the criteria were retained equally problematic for each gender (42).

Boggs et al. (27) evaluated in 668 individuals the presence of a sex bias in the diagnostic criteria for four personality disorders: borderline, schizotypal, avoidant, and obsessive-compulsive personality disorders. Authors adopted a regression model to identify bias as differences between males and females across each diagnostic criterion and level of functioning. In contrast with the other diagnoses that were examined in this study, the BPD criteria showed the largest functional disparity between genders. Only the criterion “impulsivity in multiple areas” did not display a bias among genders. These findings highlighted that all BPD criteria, except for impulsivity, tended to underestimate the level of global functioning in women as compared to men (27).

In recent years, Authors have attempted to explain whether the gender differences in prevalence of BPD is due to true group differences or to a diagnostic bias using a different and specific method: “Item response theory” (IRT) to detect “Differential Item Functioning” (DIF) (43). DIF occurs “when individuals who have the same standing on the latent trait do not have the same probability of item endorsement” (44, 45). DIF is only identified after checking for group differences on the latent trait. Thus it can be adopted as a powerful method to settle true group differences from bias at the level of DSM criterion (43).

Jane et al. (38) checked DSM-IV personality disorder criteria for potential DIF in a nonclinical sample with a rather equal distribution among gender (433 military participants and 166 college students) in which only 12 and 3 individuals in each sample evidenced BPD traits. Authors found that none of the BPD criteria demonstrated gender bias. These results could be interpreted with caution because of the limited presence of BPD traits detected in the overall sample (38).

Sharp et al. (43) evaluated gender-based DIF in BPD criteria in a sample of 747 adult psychiatric inpatient (376 males and 371 females). For the majority of criteria (seven out of nine) authors found a gender invariance, while for the criteria: “uncontrolled anger” and “impulsivity” they observed a different functioning between gender. This result suggested that SCID formulations of these criteria lead in clinicians to a greater tendency to assign positive ratings to men (43).

Finding concerning the imbalance between genders (in favor of males) of the assignment of the criterion “impulsivity” was confirmed in the study performed by Hoertel et al. (46). These authors evaluated data from the second wave of the National Epidemiologic Survey on Alcohol and Related Conditions (NESARC), including 34.653 subjects. Females and males were compared. Differences among genders were found for some specific BPD items. Females tended to be less prone to endorse impulsivity at lower degree of borderline personality disorder severity than males. On the opposite, affective instability, self-mutilating behaviors/suicidality and chronic feelings of emptiness were more discriminant in terms of severity in women than in men. No significant differences among genders were observed for the remaining DSM-IV symptoms.

These data were substantially confirmed in the recent study performed by Martin and et al. (47). They included 22.060 undergraduate students (65.5% females) tested with the McLean Screening Instrument for Borderline Personality Disorder. DIF was detected for: self-harm/suicidality, affective lability, abandonment, impulsivity, and anger. At the same level of the latent construct of BPD, females were more likely to endorse self-harm behaviors/suicidality, affective instability, and fears of abandonment, while males were more likely to endorse anger and impulsivity at lower levels of BPD.

Although studies having specifically investigated the possibility that the imbalance in the prevalence of BPD between males and females depends on diagnostic biases are still very limited, it is possible to make some comments. Studies are rather heterogeneous: some investigations were conducted in non-clinical samples (14, 38, 41, 47, 48) and one study was focused on the misdiagnosis of BPD when the physician and the patient were of the same gender (41).

One investigation reported that all diagnostic criteria display a bias, except for impulsivity (27), while another study stated that none of the BPD criteria demonstrated a gender bias (38). More recent studies (43, 46, 47) substantially agree in retaining that gender bias in diagnosis exists. In particular, at the same level of the latent traits of BPD, men were more likely to endorse intense and inappropriate anger and impulsivity (43, 47), whereas women were more likely to endorse chronic feelings of emptiness, affective instability, and suicidality/self-harm behaviors (46, 47). Results are reported in Table 1.

**Table 1:** Gender differences in the diagnosis of BPD.

Authors	Study design	States	Sample characteristics	Outcomes
Boggs et al. (2005) (27)	Longitudinal Study (Collaborative Longitudinal Personality Disorders Study)	United States	*N* = 668 individuals ♀ 64% ♂ 36%	BPD criteria, except for impulsivity, tended to underestimate the level of global functioning in ♀ as compared to ♂
Henry and Cohen (1983) (14)	Cross sectional	United States	*N* = 277 undergraduate and graduate students ♀ 197 ♂ 80	Healthy ♂ > healthy ♀ (BPD characteristics)
Hoertel et al. (2014) (45)	Epidemiologic survey Second wave of the National Epidemiologic Survey on Alcohol and Related Conditions (NESARC)	United States	*N* = 34.653 subjects ♀ 19,984 ♂ 14,497	♀ < ♂ impulsivity at lower degree of borderline personality disorder severity ♀ > ♂affective instability, self-mutilating behavios/suicidality and chronic feelings of emptiness.
Jane et al. (2007) (38)	Cross sectional study	United States	*N* = 599 subjects ♀ 284 ♂ 315	None of the BPD criteria demonstrated gender bias
Martin et al. (2023) (46)	Cross sectional study	France	*N* = 22,060 undergraduate students (females) ♀ 64.9% ♂ 35.1%	♀ > ♂ self-harm/suicidality, affective lability, abandonment ♀ < ♂ impulsivity, and anger
Morey and Ochoa (1989) (41)	Cross sectional study	United States	*N* = 291 patients *N* = 101 psychiatrists +190 psychologists	BPD ♀ were more likely to be misdiagnosed with BPD when both the physician and the patient were females
Morey et al. (2002) (42)	Cross sectional study	United States	*N* = 101 male and female college students	No gender differences in the self-ratings of BPD criteria and the criteria were retained equally problematic for each gender
Sharp et al. (2014) (43)	Cross sectional study	United States	*N* = 747 psychiatric inpatient ♀ 371 ♂ 376	♂ > ♀ uncontrolled anger and impulsivity criteria

BPD, borderline personality disorder; ♂, men; ♀, women.

## 3 The role of gender in temperamental and clinical characteristics of BPD

Several research groups have examined gender differences in BPD, but they have mainly focused on psychiatric comorbidities (see the following paragraph) and rather little attention has been paid to the differences among genders in temperamental and personality traits and in understanding how males and females express the disease clinically. Referring to Cloninger’s psychobiological model, some authors suggested that differences exist in temperament dimensions between genders (15, 49), particularly in novelty seeking, the temperament dimension that specifically differentiated BPD patients from non-clinical patients, patients without personality disorders, and patients with other personality disorders (including other diagnoses of cluster B disorders). Study performed by Barnow et al. (15) comparing a large inpatient sample of 202 subjects with BPD with other clinical control groups stated that explosive elements with high level of novelty seeking appear to differentiate men from women with BPD. In fact, BPD males were characterized by high levels of novelty seeking and harm avoidance, while BPD females had high levels of harm avoidance, but not of novelty seeking (15). Banzhaf et al. (49) evaluated the dimensional personality profiles of men and woman in 171 BPD patients. They found only few differences in personality profiles and clinical manifestations: females displayed a higher rate of neuroticism and agreeableness, while males showed higher rates of dissocial behaviors. These findings are consistent with the sex role theories regarding affect regulation, which suggest that women use more self-focused activities with internalizing behaviors than men (4, 49). Among temperamental traits, aggression was found a predictor for the early diagnosis of BPD with some gender differences in a prospective study performed in 484 adolescents with BPD features (50). In particular, relational aggression was the main predictor in boys, while physical aggression was the strongest predictor in girls.

Concerning clinical characteristics, authors do not all agree in retaining that symptoms and severity of BPD are meaningfully different in the two sexes (10, 13). For example, Johnson et al. (13) concluded that men and women “displayed more similarities than differences” (13). Zlotnick (12) did not find different level of emotional distress or overall impairment at clinical presentation in BPD females compared with BPD males. Marchetto and co-workers (2006) sustained that there may not be gender differences in BPD with regard to specific kind of self-harm conducts (i.e., self-cutting) (10).

Nevertheless, several investigations showed that distinctive symptom patterns among genders exist (3–9). McCormick et al. (4) enrolled 163 BPD patients (138 females and 25 males) to evaluate specific symptom domains in males and females. They found few differences in overall severity of symptoms, but women showed higher levels of depression, anxiety, and obsessive-compulsive symptoms than males (4). Some of these findings were confirmed by a large community sample of 6.838 individuals with a more equal distribution of gender (3,287 males and 3,551 females) (5). Authors investigated sex differences in the features of BPD, assessed with the Personality Assessment Inventory–Borderline Features scale (PAI-BOR) (51). Women reported more borderline characteristics for affective instability (depression and anxiety), disturbed relationships, but not for self-harm. An interesting result concerns identity disturbance. Women seemed to experience and report more frequent problems in the area of identity. This finding had already emerged from the study of Johnson et al. (13) in which more females endorsed the criterion “identity disturbance” than males at the Diagnostic Interview for DSM-IV Personality Disorders (DIPD-IV) (13). Tadićc et al. (6) performed a study in 159 BPD patients (110 women and 49 men) and found that women with BPD experienced more often affective instability than men, who displayed more frequent outbursts of anger (6). A relevant contribution comes from a 11-years follow-up study (3) that combines the results of two major studies: the Avon Longitudinal Study of Parents and Children (ALSPAC) and the National Epidemiologic Survey on Alcohol and Related Conditions (NESARC) (2004). This investigation had two objectives (1): to assess the prevalence of DSM-IV borderline personality disorder and its constituent symptoms in a community sample of late-latency children (6.330 subjects), and (2) to compare these rates to those found in a community sample of American adults (34.653 subjects). The resulting comparison of the ALSPAC data and the NESARC data showed that significant gender differences in clinical manifestations of BPD were found in childhood as well as in adulthood. In particular, in females a higher level of mood reactivity and chronic feelings of emptiness was registered, while males were observed to have engaged in at least two forms of impulsivity other than self-destructive acts.

Regarding the symptom dimension of aggression, the issue is controversial. In fact, some Authors retained that there are not differences in prevalence of aggression between men and women with BPD. No gender differences in aggression of BPD patients were found by Newhill et al. (37) in a sample of 220 patients (116 females and 104 males), by Scott et al. (52) in 75 psychiatric outpatients and 75 community residents (98 females and 52 males), and by Silberschmidt and co-workers (2015) in 770 patients (559 females and 211 males) (52). Moreover, in the last study, women displayed a higher degree of hostility than men. In contrast with these results, Sher et al. (8) found that men with BPD were more aggressive than women with BPD. Authors specified that the difference concerned physical aggression, but not verbal aggression (8).

Finally, a study focusing on gender differences in 207 BPD patients (140 females and 67 males) in terms of emotional and cognitive dimensions, namely hopelessness, alexithymia, sensory profile, and coping strategies (9), showed that women had higher levels of alexithymia and hopelessness in comparison with men, and higher levels of sensory sensitivity. Concerning coping strategies females displayed a higher degree of “restraint coping” and “use of instrumental social support” than males.

In summary, available investigations suggested statistically significant but modest differences among gender in terms of clinical manifestations and symptom domains. BPD females more likely reported affective instability (3–6), identity disturbance (5, 13), chronic feelings of emptiness (3), and unstable relationships (5), but not self-harm behaviors (3, 5, 10). Conversely, men were significantly more likely to engage in impulsive behaviors (3) and express outbursts of anger (6). Females with BPD showed hypersensitivity to external stimuli, which may generate anxiety and avoidance, and had higher levels of alexithymia with a greater tendency to despair (9). Taken together, results regarding aggression suggested that BPD attenuates rather than aggravates the gender difference in aggression usually present in the general population (53). Results are reported in Table 2.

**Table 2:** The role of gender in temperamental and clinical characteristics of BPD.

Authors	Study design	States	Sample characteristics	Outcomes
Amerio et al. (2023) (9)	Cross sectional	Italy	*N* = 207 BPD patients ♀ 140 ♂ 67	♀ > ♂ alexithymia, hopelessness and sensory sensitivity ♀ > ♂ *restraint coping* and *use of instrumental social support*
Banzhaf et al. (2012) (49)	Cross sectional	Germany	*N* = 171 BPD patients ♀114 ♂56	♂ dissocial behaviors ♀ neuroticism and agreeableness
Barnow et al. (2007) (15)	Cross sectional	Germany	*N* = 202 BPD patients ♀ 67.3% ♂32.7%	♂ BPD: novelty seeking and harm avoidance ♀ BPD: harm avoidance, but not novelty seeking
De Moor et al. (2009) (5)	Cross sectional	Netherlands	*N* = 6.838 subjects ♀ 3,551 ♂ 3,287	♀ > ♂ borderline characteristics for affective instability (depression and anxiety), disturbed relationships, but not for self-harm ♀ > ♂ identity disturbance
Johnson et al. (2003) (13)	Cross sectional	United States	N = 240 ♀175 ♂65	Men and women displayed more similarities than differences ♀ > ♂ identity disturbance
Marchetto et al. (2006) (10)	Cross sectional	United Kingdom	*N* = 516 ♀250 ♂266	No gender differences in BPD with regard to specific kind of self-harm conducts
McCormick et al. (2007) (4)	Cross sectional	United States	*N* = 163 BPD patients ♀138 ♂ 25	♀ > ♂ depression, anxiety, and obsessive-compulsive
Newhill et al., 2009 (37)	Longitudinal study	United States	*N* = 220 BPD patients ♀ 116 ♂ 104	No gender differences in aggression of BPD patients
Scott et al. (2014) (52)	Longitudinal study	United States	*N* = 75 psychiatric outpatients +75 community residents ♀ 98 ♂ 52	No gender differences in aggression of BPD patients
Sher et al. (2019) (8)	Cross sectional	United States	*N* = 511 BPD patients ♀ 203 ♂ 145	♀ < ♂ physical aggression
Silberschmidt et al. (2015) (7)	Cross sectional	United States	*N* = 770 patients ♀ 559 ♂ 211 males	No gender differences in aggression of BPD patients ♀ > ♂ hostility
Tadić et al. (2009) (6)	Cross sectional	Germany	*N* = 159 BPD patients ♀ 110 ♂ 49	♀ > ♂ affective instability ♀ < ♂outbursts of anger
Vaillancourt et al. (2014) (50)	Cross sectional	Canada	*N* = 484 adolescents with BPD features ♀ 55% ♂ 45%	Aggression was found a predictor for the early diagnosis of BPD: ♂ relational aggression ♀ physical aggression
Zanarini et al. (2011) (3)	Longitudinal study	United States	*N* = 6.330 adolescent subjects ♀ 3,273 ♂ 3,057 *N* = 34,653 adults subjects ♀ 14,564 ♂ 20,089	♀ > ♂ higher level of mood reactivity and chronic feelings of emptiness ♀ < ♂ impulsivity other than self-destructive acts
Zlotnick et al. (2002) (12)	Cross sectional	United States	*N* = 139 ♀105 ♂44	No different level of emotional distress or overall impairment at clinical presentation in BPD ♀ compared with BPD ♂

BPD, borderline personality disorder; ♂, men; ♀, women.

## 4 Comorbidities and gender

The presence of two or more psychiatric conditions in an individual is a complex phenomenon that can vary on the basis of several factors, including gender. BPD is not an exception. As we will illustrate in this section, there are notable differences in comorbidity patterns between males and females with BPD.

A first major scientific contribution to the study of gender differences in comorbidity in BPD patients was provided by Zanarini and et al. (54, 55), who conducted two studies involving a sample of 504 patients. The aim of the studies was to assess the lifetime rates of occurrence of a full range of DSM-III-R axis I (54) and axis II (55) disorders in a group of patients diagnosed with BPD. Concerning Axis I disorders (54), Authors showed that male and female BPD patients had similar rates of comorbidity in terms of psychotic disorders (about 1%), somatoform disorders (about 10%), anxiety disorders (over 80%), and mood disorders (over 90%). In contrast, a higher percentage of male BPD patients fulfilled the criteria for alcohol abuse/dependence (74% male versus 46% female), drug abuse/dependence (65% male versus 41% female), and overall substance abuse/dependence (82% male versus 59% female). On the opposite, a considerably greater proportion of BPD patients who were females met the criteria for anorexia nervosa (25% females versus 7% males), bulimia nervosa (30% females versus 10% males), eating disorder not otherwise specified (almost all cases were either binge eating disorder or purging disorder) (30% females versus 11% males), as well as the overall eating disorder category (62% females versus 21% males). Women with BPD were notably more likely than men to fulfill the DSM-III-R criteria for post-traumatic stress disorder (PTSD) (61% females versus 35% males). Several following studies reported similar results stating that females with BPD more often met the criteria for PTSD (6, 7, 13, 16, 49, 56), and eating disorders (6, 7, 12, 13, 56). Some authors reported significant differences also for major depression disorder (6, 7, 16), and anxiety disorders (4, 49). As concern substance use disorder, many studies confirm that it seems to be more common in men than in women (6–8, 12, 13).

However, some studies provide data not concordant with more common findings. McCormick et al. (4) did not find differences among genders in the frequency of substance use disorders, depressive disorders, PTSD, panic disorder, and anorexia nervosa. It may be due to a different method in selection of the samples, choice of inclusion and exclusion criteria, and assessment of patients (4).

More recently, Dehlbom et al. (56) observed that men with BPD presented more often non-affective psychosis than woman (respectively 10.7% versus 5.9%).

As concerns Axis II disorders comorbidities, Zanarini et al. (55) highlighted some significant differences between male and female BPD patients: a significantly higher percentage of males than females met DSMIII-R criteria for narcissistic personality disorder (respectively 30% vs. 13%), antisocial personality disorder (respectively 48% vs. 16%), paranoid personality disorder (respectively 45% vs. 26%), passive-aggressive personality disorder (respectively 46% vs. 19%), and sadistic personality disorder (respectively 16% vs. 3%).

Some of these data were confirmed by other investigations. In particular, men with BPD were found to present higher rates than women of antisocial personality disorder (4, 6–8, 13, 16, 49, 56, 57), narcissistic personality disorder (7, 8, 13, 16, 49), and schizotypal personality disorders (8, 13).

On the other hand, women showed more frequently than men histrionic personality disorder (4, 6), dependent personality disorder (8, 57), and obsessive-compulsive personality disorder (8).

Regarding comorbidity with PTSD and exposure to trauma a specification is needed. Some symptoms of complex-PTSD are overlapping with those of BPD. Both disorders are characterized by difficulties in affect regulation, self-concept, and interpersonal relationships (58). As a result, we cannot rule out that some patients receive a diagnosis of BPD when in fact complex-PTSD better fits their experiences (59). This misdiagnosis may contribute to the difference in prevalence between genders since complex-PTSD as well as PTSD has been found more prevalent in females (60, 61).

In conclusion, we can suggest that BPD women more often experience “internalizing” disorders in comorbidity with BPD, while men more often report “externalizing” disorders. As hypothesized by Krueger (62), internalizing disorders reflect inwardly directed distress. Thus patients are more likely to meet the criteria for depression, anxiety, eating disorders, and somatoform disorders. On the other hand, externalizing disorders are directed outwardly, placing the subject at odds with society, as it happens in antisocial personality disorder or substance use disorder. Results are shown in Table 3.

**Table 3:** Comorbidities and gender.

Authors	Study design	States	Sample characteristics	Outcomes
Banzhaf et al. (2012) (49)	Cross sectional	Germany	*N* = 171 BPD patients ♀ 114 ♂ 57	Bulimia nervosa: 19.3% ♀ vs. 5.36% ♂ Binge eating disorder: 0.87% ♀ vs. 7.14% ♂ Panic with agoraphobia: 9.6% ♀ vs. 3.6% ♂ PTSD: 35.96% ♀ vs. 14.29% ♂ Antisocial personality disorder: 10.53% ♀ vs. 32.14% ♂ Narcissistic personality disorder 2.63% ♀ vs. 20.45% ♂
Barrachina et al. (2011) (57)	Cross sectional	Spain	*N* = 484 BPD patients ♀ 402 ♂ 82	Antisocial personality disorder: 8.2% ♀ vs. 22% ♂ Dependent personality disorder 2.4% ♀ vs. 17.2% ♂
Dehlbom et al. (2022) (56)	Cross sectional	Sweden	*N* = 5,530 BPD patients ♀ 4,728 ♂ 802	Antisocial personality disorder: 1.1% ♀ vs. 6.7% ♂ PTSD 16.5% ♀ vs. 11.2% ♂ Eating disorder: 15.1% ♀ vs. 3.1% ♂ Substance use disorders: 34.2% ♀ vs. 49.3% ♂ Nonaffective psychosis: 5.9% ♀ vs. 10.7% ♂
Grant et al. (2008) (16)	Epidemiologic survey Second wave of the National Epidemiologic Survey on Alcohol and Related Conditions (NESARC)	United States	*N* = 34,653 subjects; ♀ 6.2% ♂ 5.6%	Major depressive disorder 36.1% ♀ vs. 27.2%♂ Dysthymia 11.9% ♀ vs. 7.1%♂ Any substance use disorder 66.2%♀ vs. 80.9% ♂ Panic with agoraphobia 9.6% ♀ vs. 3.6% ♂ Specific phobia 31.7% ♀ vs. 16.2% ♂ Generalized anxiety 26.4% ♀ vs. 18.7% ♂ PTSD 38.2% ♀ vs. 23.6% ♂ Antisocial personality disorder: 9% ♀ vs. 19.4% ♂ Narcissistic personality disorder 32.2% ♀ vs. 47.0% ♂
Johnson et al. (2003) (13)	Cross sectional	United States	*N* = 668 PD patients; among them 240 BPD patients ♀ 175 ♂ 65	Substance use disorders: 58.3% ♀ vs. 84.6% ♂ PTSD: 50.9% ♀ vs. 30.8% ♂ Eating disorder: 41.7% ♀ vs. 18.5% ♂ Narcissistic personality disorder 4.6% ♀ vs. 21.9% ♂ Antisocial personality disorder: 10.3% ♀ vs. 29.7% ♂ Schizotypal personality disorder: 10.3% ♀ vs. 24.6% ♂
McCormick (2007) (4)	Cross sectional	United States	*N* = 163 BPD patients ♀ 138 ♂ 25	Antisocial personality disorder: 21% ♀ vs. 40% ♂ Generalized anxiety disorder: 55% ♀ vs. 32% ♂ Any somatoform disorder: 14% ♀ vs. 0% ♂ Histrionic personality disorder: 25% ♀ vs. 0% ♂
Sher et al. (2019) (8)	Case–Control study	United States	N = 348 BPD patients ♀ 203 ♂145 *N* = 163 HC ♀82 ♂ 81	Narcissistic personality disorder 36% ♀ vs. 64.1% ♂ Antisocial personality disorder: 16.7% ♀ vs. 55.2% ♂ Schizotypal personality disorder: 21.7% ♀ vs. 34.5% ♂ Paranoid personality disorder: 30.5% ♀ vs. 49.7% ♂ Alcohol and substance use disorders: 36.9% ♀ vs. 53.1% ♂ Dependent personality disorder: 25.6% ♀ vs. 15.9% ♂ Obsessive-compulsive personality disorders: 60.1% ♀ vs. 46.9% ♂
Tadić et al. (2009) (6)	Cross sectional	Germany	*N* = 159 BPD patients ♀ 110 ♂ 49	Substance use disorders: 67.3% ♀ vs. 83.7% ♂ Alcohol dependency 42.7% ♀ vs. 65.3% ♂ Affective disorder: 94% ♀ vs. 82% ♂ Anxiety disorder: 92% ♀ vs. 80% ♂ Eating disorder: 35% ♀ vs. 18% ♂ Anorexia nervosa: 21% ♀ vs. 4% ♂
Zanarini et al. (1998a) (54)	Cross sectional	United States	*N* = 504 patients; *N* = 379 with BPD diagnosis ♀ 296 ♂ 83 *N* = 125 other PD diagnosis ♀ 70 ♂ 55	Substance abuse: alcohol abuse/dependence: 46% ♀ vs. 74% ♂ drug abuse/dependence: 41% ♀ vs. 65% ♂ overall substance abuse/dependence: 59% ♀ vs. 82% ♂ ED: anorexia nervosa: 25% ♀ vs. 7% ♂ bulimia nervosa: 30% ♀ vs. 10% ♂ eating disorder not otherwise specified: 30% ♀ vs. 11% ♂ overall eating disorder category: 62% ♀ vs. 21% ♂ PTSD: 61% ♀ vs. 35% ♂
Zanarini et al. (1998b) (55)	Cross sectional	United States	*N* = 504 patients; *N* = 379 with BPD diagnosis ♀ 296 ♂ 83 *N* = 125 other PD diagnosis ♀ 70 ♂ 55	Narcissistic personality disorder 13% ♀ vs. 30% ♂ Antisocial personality disorder: 16% ♀ vs. 48% ♂ Paranoid personality disorder: 26% ♀ vs. 45% ♂ Passive-aggressive personality disorder: 19% ♀ vs. 46% ♂ Sadistic personality disorder 3% ♀ vs. 16% ♂ Cluster: odd cluster disorder: 27% ♀ vs. 47% ♂ dramatic cluster disorder: 32% ♀ vs. 68% ♂
Zlotnick et al. (2002) (12)	Cross sectional	United States	*N* = 1,500 outpatients; *N* = 149 BPD patients ♀ 105 ♂ 44	Eating disorder: 29.5% ♀ vs. 13.6% ♂ Substance use disorder: 38.1% ♀ vs. 63.6% ♂ Antisocial personality disorder 11.4% ♀ vs. 38.6% ♂

BPD, borderline personality disorder; HC, Healthy Controls; PTSD, Post-Traumatic Stress Disorder; ED, Eating Disorders; ♂, men; ♀, women.

## 5 Gender differences and neuroimaging in BPD

While BPD is primarily diagnosed through clinical assessment and criteria defined in the Diagnostic and Statistical Manual of Mental Disorders (DSM-5), neuroimaging research aims to shed light on the brain mechanisms and structural/functional differences associated with the disorder. Within this framework, we analyzed the limited and preliminary data suggesting differences in neuroimaging findings between male and female individuals with BPD. It is necessary to note that, to our knowledge, there are no studies that have specifically assessed structural brain differences in BPD by direct gender comparisons. The observations we can draw derive from studies that have evaluated women with BPD versus healthy women and men with BPD versus healthy subjects of the same gender. Some studies suggested that there may be gender differences in the structural brain abnormalities associated with BPD. Soloff et al. (63) in a study on 34 BPD subjects (22 females, 12 males) and 30 healthy controls (19 females and 11 males) distinguished gender-specific neuroanatomical differences. They observed that, in contrast to healthy controls of the same gender, women with BPD exhibited decreased gray matter volumes in the amygdala and hippocampus, whereas men with BPD displayed decreased volume in the anterior cingulate cortex and increased volume in the right putamen (63). These data are aligned with findings from two studies focused exclusively on male BPD patients compared to healthy males. Volume reductions were found in specific brain regions, including the superior, medial, and middle frontal gyrus (64), as well as the orbital frontal cortex and ventromedial prefrontal cortex (65). Divergent findings were obtained by Mancke et al. (66), that analyzed data from 21 male BPD patients and 51 healthy controls. Authors observed no differences in amygdala volume between BPD patients and healthy volunteers, but a trend for a positive association between volume of the right amygdala and aggressiveness in male BPD patients and, through analyses of amygdala shape, a positive association between regions of the left superficial and laterobasal amygdala of male BPD patients and aggressiveness.

With regard to functional neuroimaging, five investigations evaluated the abnormalities of brain activity and connectivity in men and women with BPD (67–71).

The study by New et al. (67) on 38 BPD patients with intermittent explosive disorder and 36 healthy controls who participated in the Point Subtraction Aggression Paradigm (PSAP), a validated method of provoking aggression in a laboratory setting through a computer game, did not found any gender differences in brain metabolism in prefrontal and amygdala areas while participants were performing the PSAP. Patients reacted aggressively and showed increased activity in emotional brain areas, including amygdala and orbital frontal cortex in response to provocation, but not in more dorsal brain regions associated with cognitive control of aggression. In contrast, healthy controls showed an increased activity in dorsal regions of prefrontal cortex, that are areas involved in top-down cognitive control of aggression and, more broadly, of emotion (67).

In a following analysis of the same data, conducted by Perez-Rodriguez et al. (70), authors focused on the role of the corpus striatum, which is closely connected with the medial frontal cortex and plays an important role in motivated behaviors and in the processing of rewarding stimuli (72). In this analysis, some gender differences emerged. In comparison to female patients, males with BPD and concurrent Intermittent Explosive Disorder exhibited a notably reduced glucose metabolism rate within the striatum. The decreased activity in the striatum that was observed in male BPD patients may be linked to a potential difficulty in assessing situational context. This abnormality could hinder the inhibitory functions of prefrontal regions, possibly leading to an increase in aggressive behavior over time (70).

The role of serotonin-2A receptor was also investigated by Soloff and collaborators, using positron emission tomography. Differences in serotonin-2A receptor binding were found in subjects with BPD compared to healthy subjects. Gender effects were present, as binding potentials predicted impulsivity and aggression in BPD females and healthy males, but not in BPD males. Authors hypothesized that gender may mediate the clinical expression of aggression and impulsivity in BPD (68).

A relevant contribution in this field was given by Herpertz et al. (69) who performed a functional magnetic resonance imaging study on 33 female and 23 male patients with BPD and 30 healthy women and 26 healthy men. Some specific gender differences were found: males with BPD were found to have a higher activation in the left amygdala than female patients. During the anger induction (anger induction was based on narratives of harsh interpersonal rejections) men with BPD exhibited higher activity in the lateral orbitofrontal and dorsolateral prefrontal cortices compared with healthy men and female patients. In female patients a positive connectivity between the amygdala and the posterior middle cingulate cortex was observed, while in male patients this connectivity was negative. In addition, in male patients authors showed negative modulatory effects of trait anger on the matching between the amygdala and the dorsolateral prefrontal cortex, as well as the amygdala and the lateral orbitofrontal cortex, whereas in female patients trait anger had a positive modulatory effect on the coupling between the dorsolateral prefrontal cortex and the amygdala (69). These results are in line with another trial that identified a connection of aggressiveness and trait anger with specific brain regions such as amygdala, anterior prefrontal cortex, and dorsolateral prefrontal cortex in male BPD patients (71).

More in details, Bertsch et al. enrolled 15 medication-free male patients with BPD and 25 healthy men who took part in a social Approach-Avoidance task in the magnetic resonance-scanner. Male patients with BPD showed reduced anterolateral prefrontal activations during emotional action control compared to healthy volunteers. Furthermore, outbursts of anger were negatively related to antero- and dorsolateral prefrontal activations, while they were positively related to amygdala activity in male patients.

Unfortunately, neuroimaging studies that have directly compared male and female subjects with BPD are too few to draw any conclusions. The observations that can be cautiously made are inferred from limited samples and in some cases of only one gender. The strongest finding that emerges from investigations that we have here discussed, is that there are some gender differences in activity of prefrontal cortex, striatum, and amygdala when patients are subjected to fMRI tasks that induce anger/aggression reactions. Results are displayed in Table 4.

**Table 4:** Gender differences and neuroimaging in BPD.

Authors	Study design	States	Sample Characteristics	Outcomes
Bertsch et al. (2013) (65)	Case–control study	Germany	*N* = 39 ♂ BPD patients with comorbid ASPD (BPD-ASPD) *N* = 14 ♂ HC	♂ BPD patients displayed volume reduction in the left frontal pole, left orbital frontal cortex and right ventromedial prefrontal cortex compared to ♂ healthy controls
Bertsch et al. (2019) (71)	Case–control study	Germany	*N* = 15 medication-free ♂ BPD patients *N* = 25 ♂ HC	BPD patients showed reduced anterolateral prefrontal activations during emotional action control compared to healthy volunteers. Furthermore, outbursts of anger were negatively related to antero- and dorsolateral prefrontal activations, while they were positively related to amygdala activity in patients.
Herpertz et al. (2017) (69)	Case–control study	Germany	*N* = 56 BPD patients ♀ 33 ♂ 23 *N* = 56 HC ♀ 30 ♂ 26	♂ BPD patients revealed higher activity in the left amygdala than ♀ patients; furthermore ♂ BPD showed higher activity in the lateral orbitofrontal and dorsolateral prefrontal cortices compared with ♂ HC and ♀ patients. ♀ patients highlight a positive connectivity between the amygdala and the posterior middle cingulate cortex was observed, while in ♂ patients this connectivity was negative. In ♂ patients there are negative modulatory effects of trait anger on the matching between the amygdala and the dorsolateral prefrontal cortex, as well as the amygdala and the lateral orbitofrontal cortex, whereas in ♀ patients trait anger had a positive modulatory effect on the coupling between the dorsolateral prefrontal cortex and the amygdala.
Mancke et al. (2018) (66)	Case–control study	Germany	*N* = 58 BPD patients ♀ 37 ♂ 21 *N* = 51 HC ♀ 28 ♂ 23	No differences in amygdala volume were found between BPD patients and healthy volunteers. ♂ BPD patients showed a positive association between volume of the right amygdala with aggressiveness, and between regions of the left superficial and laterobasal amygdala with aggressiveness.
New et al. (2009) (67)	Case–control study	United States	*N* = 38 BPD patients with intermittent explosive disorder (BPD-IED) comorbid ♀ 16 ♂ 22 *N* = 36 HC ♀ 18 ♂ 18	Patients showed increased relative glucose metabolic rate (rGMR) in emotional brain areas, including amygdala and orbitofrontal cortex (OFC), in response to provocation, but not in more dorsal brain regions associated with cognitive control of aggression. In contrast, HC increased rGMR in dorsal regions of prefrontal cortex (PFC), involved in top-down cognitive control of aggression and emotions.
Perez-Rodriguez et al. (2012) (70)	Case–control study	United States	*N* = 38 BPD patients with intermittent explosive disorder (BPD-IED) comorbid ♀ 16 ♂ 22 N = 36 HC ♀ 18 ♂ 18	♂ BPD displayed reduced glucose metabolism rate in the striatum when performing the Point Subtraction Aggression Paradigm (PSAP) compared to ♀ BPD and HC of both gender (*p* < 0.01). No gender differences in prefrontal and amygdala regions.
Soloff et al. (2008) (63)	Case–control study	United States	*N* = 34 BPD patients ♀ 22 ♂ 12 (among them 2 ♀ BPD-ASPD and 7 ♂ BPD-ASPD) *N* = 30 HC ♀ 19 ♂ 11	♀, but not ♂, BPD patients showed a gray volume reduction the amygdala, hippocampus bilaterally compared to healthy controls ♂, but not ♀ BPD patients showed a gray matter volume reduction in the anterior cingulate cortex and a gray matter volume increase in the right putamen when compared to healthy controls
Soloff et al. (2014) (68)	Case–control study	United States	*N* = 33 BPD patients ♀ 20 ♂ 13 *N* = 27 HC ♀ 12 ♂ 15	Differences in serotonin-2A receptor binding were found in subjects with BPD compared to healthy subjects. Binding potentials predicted impulsivity and aggression in BPD females and healthy males, but not in ♂ BPD patients and ♀ HC.
Völlm et al. (2009) (64)	Case–control study	Australia	*N* = 7 ♂ BPD patients *N* = 6 ♂ HC	♂ BPD patients showed gray matter volume reduction in the medial, middle and superior frontal gyrus, bilaterally, and the left orbitofrontal cortex and right anteriorcingulate cortex compared to ♂ healthy controls

BPD, borderline personality disorder; ASPD, Antisocial personality disorder; HC, Healthy Controls; ♂, men; ♀, women.

## 6 Gender differences in treatment utilization

Data about patterns of treatment utilization by gender in BPD are rather scarce. Systematic reviews performed by the Cochrane Collaboration on treatment of BPD (22, 73–75) stated that the majority of studies consisted predominantly of females (58–96%). Thus the findings may not be automatically applied to the male patients.

There is a global consensus in retaining that patients with BPD, both men and women, have elevated rates of treatment use in comparison with other clinical groups (i.e., mood and anxiety disorders and other personality disorders) (76–78).

Engagement in treatment seemed to be different between genders: men are less prone to be engaged in all types of treatment (medical or psychiatric), while women are more likely to seek help (79). A possible explanation of these differences could be that men have a tendency to exhibit a “wait-and-see” behavior (80), while women more often ask for help for psychological distress and emotional difficulties and seek consultations with less shame (81). To our knowledge, four studies reported specific findings concerning differences in treatment utilization among genders in BPD patients (4, 56, 76, 82). Investigation performed by McCormick et al. (4) was already described in the previous paragraphs. Authors did not find significant differences in the use of mental health services or in the propensity to psychiatric medication and psychotherapeutic interventions (4). These results were substantially confirmed by Tomko et al. (82) in a cross-sectional study with a community BPD sample (1.030 subjects of which 57% female). Authors reported that although female with BPD showed slightly higher rates of psychiatric treatment utilization than males (medication, visits, and hospitalizations) this finding did not reach the statistical significance (82). A following study in a larger sample (409 women and 86 men) of BPD patients verified the lifetime patterns of treatments utilization by gender using an online questionnaire filled out by patients’ relatives. Rates of lifetime use of mental health services in terms of hospitalizations, day programs and halfway houses were similar across genders. Nevertheless, male subjects showed higher levels of use of drug/alcohol rehabilitation services and they received less psychotherapeutic and pharmacological interventions in comparison with females. In addition, patterns of duration of pharmacotherapy and psychotherapy in patients who actually began treatment were similar for the two genders, with the exception of anti-anxiety medications, that were prevalent in women (76). More recently, Dehlbom et al. (56) identified 802 men and 4.728 women with BPD in the Swedish health and administrative registers and evaluated the use of mental health care (including receipt of medications and psychotherapies) together with other demographical and clinical information (56). Consistently with results from Goodman et al. (76), authors found that men received less pharmacological and psychological treatments than women. However, similarly to the Tomko’s study, most of the differences in treatment with psychological interventions were nonsignificant in a multivariate model, suggesting that they were probably due to the differences in sociodemographic variables and comorbidities between the two genders. In fact, in this sample men were older than women, with a lower level of education, and were likely to receive social support.

In conclusion, studies on gender differences in the treatment of BPD are still very limited and suffer from common limitations: sample sizes are small (4) or unbalanced in favor of the female gender (56, 76); results of one study were based solely on parental reports (76); findings for specific psychological treatments or specific psychiatric medications have not been reported (82). Based on these observations, no final conclusions can be drawn. It seems that the rates of lifetime use of mental health services are similar across genders, but males showed higher levels of use of drug/alcohol rehabilitation services and received less psychotherapeutic and pharmacological interventions compared with females. Results are reported in Table 5.

**Table 5:** Gender differences in treatment utilization.

Authors	Study design	States	Sample characteristics	Outcomes
Dehlbom et al. (2022) (56)	Cross sectional study	Sweden	*N* = 5,530 BPD patients ♀ 4.728 ♂ 802	♀ > ♂ pharmacological and psychological treatments
Goodman et al. (2010) (76)	Cross sectional study	United States	*N* = 495 BPD patients ♀ 409 ♂ 86	♀ < ♂ levels of use of drug/alcohol rehabilitation services ♀ > ♂ psychotherapeutic and pharmacological interventions.
McCormick et al. (2007) (4)	Cross sectional study	United States	*N* = 163 BPD patients ♀ 138 ♂ 25	No significant differences in the use of mental health services or in the propensity to psychiatric medication and psychotherapeutic interventions.
Tomko et al. (2014) (82)	Epidemiologic survey Second wave of the National Epidemiologic Survey on Alcohol and Related Conditions (NESARC)	United States	*N* = 34,481 subjects ♀ 19,984 ♂ 14,497	♀ > ♂ psychiatric treatment utilization (not statistical significance)

BPD, borderline personality disorder; ♂, men; ♀, women.

## 7 Conclusion

Studies that have specifically assessed gender differences in BPD are still scarce and designed with heterogeneous methods (e.g., some studies regarding the fulfillment of diagnostic criteria for BPD were conducted in samples of nonclinical populations, most studies recruited samples with a higher proportion of female subjects, gender comparisons are inferred from comparisons between cases and controls of a single gender). With these limitations, the most relevant results obtained in studies of BPD patients in the last two decades are the following:

- men seemed to be more likely to endorse the DSM diagnostic criteria “intense and inappropriate anger” and “impulsivity,” whereas women were more likely to endorse the criteria “chronic feelings of emptiness,” “affective instability,” and “suicidality/self-harm behaviors”;
- higher level of the temperament dimension “novelty seeking” seemed to differentiate men from women with BPD. Moreover, BPD females showed a higher degree of affective instability, identity disturbance, chronic feelings of emptiness, and unstable relationships, but not self-harm behaviors in comparison with BPD males, while men were more engaged in impulsive behaviors and outbursts of anger than women. It is noticeable that a diagnosis of BPD appears to attenuate the gender difference in level of aggression commonly present in the general population;
- women with BPD had higher rates of comorbidity with depressive and anxiety disorders, eating disorders, and somatoform disorders. On the other hand, men were more likely to have a co-diagnosis of antisocial personality disorder or substance use disorder;
- some gender differences in activity of prefrontal cortex, striatum, and amygdala were observed when patients were tested with fMRI tasks that induced anger/aggression reactions;
- rates of use of mental health services were substantially similar among genders, but males used drug/alcohol rehabilitation services more than females and received less psychotherapeutic and pharmacotherapeutic interventions compared with females.

A review on gender differences in borderline personality disorder has been recently published (2) and deserves a comparison between its results and our findings. While conclusions concerning symptoms and comorbid disorders, developmental and biological factors, and treatment issues are mainly concordant in the two studies, our review contains a detailed examination of differences concerning prevalence of BPD and more common diagnostic criteria endorsed by male and female patients. In addition, we considered data on different profiles of temperament and personality traits. Some strengths and limitations of this review should be considered.

One of the strengths is the comprehensive comparison of the two genders regarding diagnostic criteria, manifestation, experience of symptoms and help-seeking behaviors.

Limitations include the fact that we searched only one database, we limited the time of the publications, we excluded non-English language manuscripts, and we did not provide a critique for the quality of each paper included (we did for some either individually or in a group).

Future research should be focused on implementing the knowledge that we have on this topic. In particular, if we consider gender a key element in patients assessment and management personalized interventions for males and females with BPD are a primary goal of investigations and clinical practice.

## Author contributions

PB: Conceptualization, Supervision, Writing – original draft, Writing – review & editing. CeB: Methodology, Supervision, Validation, Writing – review & editing. DB: Conceptualization, Data curation, Validation, Writing – original draft. LB: Methodology, Writing – original draft. ClB: Supervision, Writing – review & editing. PR: Supervision, Validation, Writing – review & editing. SB: Conceptualization, Methodology, Supervision, Writing – review & editing.
